# Green SPIONs as a novel highly selective treatment for leishmaniasis: an in vitro study against *Leishmania amazonensis* intracellular amastigotes

**DOI:** 10.3762/bjnano.14.73

**Published:** 2023-08-30

**Authors:** Brunno Renato Farias Verçoza, Robson Roney Bernardo, Luiz Augusto Sousa de Oliveira, Juliany Cola Fernandes Rodrigues

**Affiliations:** 1 Núcleo Multidisciplinar de Pesquisas em Biologia, NUMPEX-Bio, Campus UFRJ Duque de Caxias Prof. Geraldo Cidade, Universidade Federal do Rio de Janeiro, Rodovia Washington Luiz, n. 19593, km 104.5, 25240-005, Duque de Caxias, RJ, Brasil; 2 Núcleo Multidisciplinar de Pesquisas em Nanotecnologia, NUMPEX-Nano, Campus UFRJ Duque de Caxias Prof. Geraldo Cidade, Universidade Federal do Rio de Janeiro, Rodovia Washington Luiz, n. 19593, km 104.5, 25240-005, Duque de Caxias, RJ, Brasil

**Keywords:** coconut water, Leishmaniasis, *Leishmania amazonensis*, nanomedicine, SPIONs

## Abstract

The main goal of this work was to evaluate the therapeutic potential of green superparamagnetic iron oxide nanoparticles (SPIONs) produced with coconut water for treating cutaneous leishmaniasis caused by *Leishmania amazonensis*. Optical and electron microscopy techniques were used to evaluate the effects on cell proliferation, infectivity percentage, and ultrastructure. SPIONs were internalized by both parasite stages, randomly distributed in the cytosol and located mainly in membrane-bound compartments. The selectivity index for intracellular amastigotes was more than 240 times higher compared to current drugs used to treat the disease. The synthesized SPIONs showed promising activity against *Leishmania* and can be considered a strong candidate for a new therapeutic approach for treating leishmaniases.

## Introduction

Leishmaniasis is one of the most important neglected diseases of chronic nature and remains a serious global health problem. A worrying increase has been observed in the number of leishmaniasis cases worldwide in recent decades. It is estimated that about 600 million people live in risk areas, and 0.6–1.2 million new leishmaniasis cases appear annually [1]. The treatment for this disease involves using pentavalent antimonials, miltefosine, amphotericin B, paromomycin, or pentamidine. However, side effects of these drugs and an increased number of drug-resistant parasites have been reported [2–5]. These facts demonstrate the need to develop new treatments or alternatives that are safer, more effective, and more accessible to patients.

In this context, nanomedicine is one of the most promising branches of contemporary medicine, currently concentrating a large part of the scientific effort on the search for new treatments for different diseases. Its main objective is to develop therapies with higher specificity, effectiveness, and safety, as well as less toxicity [6]. One interesting class of nanomaterials in medicine are superparamagnetic iron oxide nanoparticles (SPIONs). SPIONs exhibit theranostic properties, that is, they can be used simultaneously for diagnosis and therapy. Thus, SPIONs have emerged as one of the best options for the development of new therapeutic methods. SPIONs offer several features such as good biocompatibility, degradability under moderate acid conditions, the ability for magnetic manipulation, the possibility of being used in magnetic resonance imaging, and the ability to generate controlled heat non-invasively when exposed to an alternating magnetic field [7–8]. In 2019, our group published an article describing a low-cost green synthesis of SPIONs using coconut water [9]. In this article, the ability of macrophages to uptake these SPIONs was evaluated, together with some physical and chemical characterizations. The synthesized green SPIONs are around 4 nm in diameter, are composed of pure nonstoichiometric magnetite, exhibit superparamagnetic behavior at room temperature, and are taken up by macrophages without being toxic for these mammalian cells [9].

The application of SPIONs in treating leishmaniasis has been studied by different groups over the past few years, showing promising and satisfactory results [10–13]; thus, using SPIONs to develop new topical treatments can mean a revolution. SPIONs could be used for topical application, associated with drugs and combined or not with thermotherapy by magnetic hyperthermia. Furthermore, the treatment can be applied to the localized cutaneous lesion, making the treatment more specific and less toxic to the patient. Thus, the main goal of this study is to evaluate the effects of green SPIONs against *Leishmania amazonensis* (*L. amazonensis*) in vitro.

## Results

### Uptake of SPIONs by *L. amazonensis* promastigotes and intracellular amastigotes

**Figure 1:**
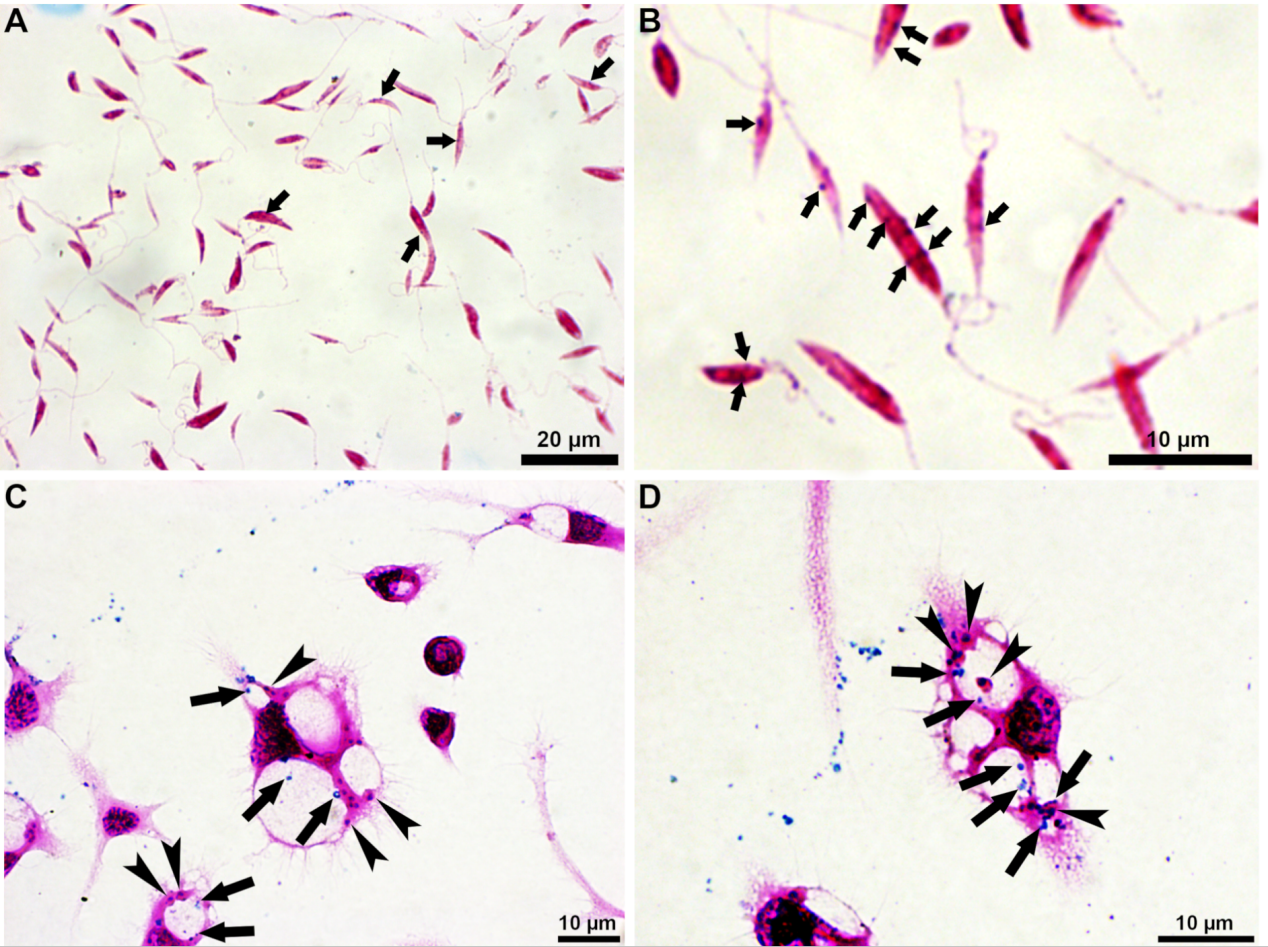
Bright-field optical microscopy of *L. amazonensis* promastigotes (A, B) and intracellular amastigotes (C, D) treated with 100 µg/mL of SPIONs for 24 h, after staining with Prussian blue (A–D). (A) The arrows indicate the blue stain characteristic for the reaction with ferrous compounds in the promastigote cytosol. (B) Digital magnification shows that SPIONs are randomly distributed throughout the cytosol. (C) In the case of macrophages infected with intracellular amastigotes, the SPIONs were observed inside the parasitophorous vacuoles. (D) Digital magnification shows the SPIONs (arrows) inside the macrophage cytosol, the parasitophorous vacuoles, and the amastigote cytosol (arrowheads).

Bright-field optical microscopy of *L. amazonensis* promastigotes and intracellular amastigotes incubated with Prussian blue revealed that both parasite stages can uptake the SPIONs (Figure 1). The arrows and arrowheads in Figure 1 show the characteristic blue stain that indicates the positive reaction between potassium ferrocyanide and ferrous compounds. In promastigotes (Figure 1A,B), the SPIONS are distributed throughout the cytosol. In contrast, in the intracellular amastigotes cultivated in macrophages, the SPIONs appear in the mammalian cytosol, inside the parasitophorous vacuole, and in the parasite cytosol (Figure 1C,D).

**Figure 2:**
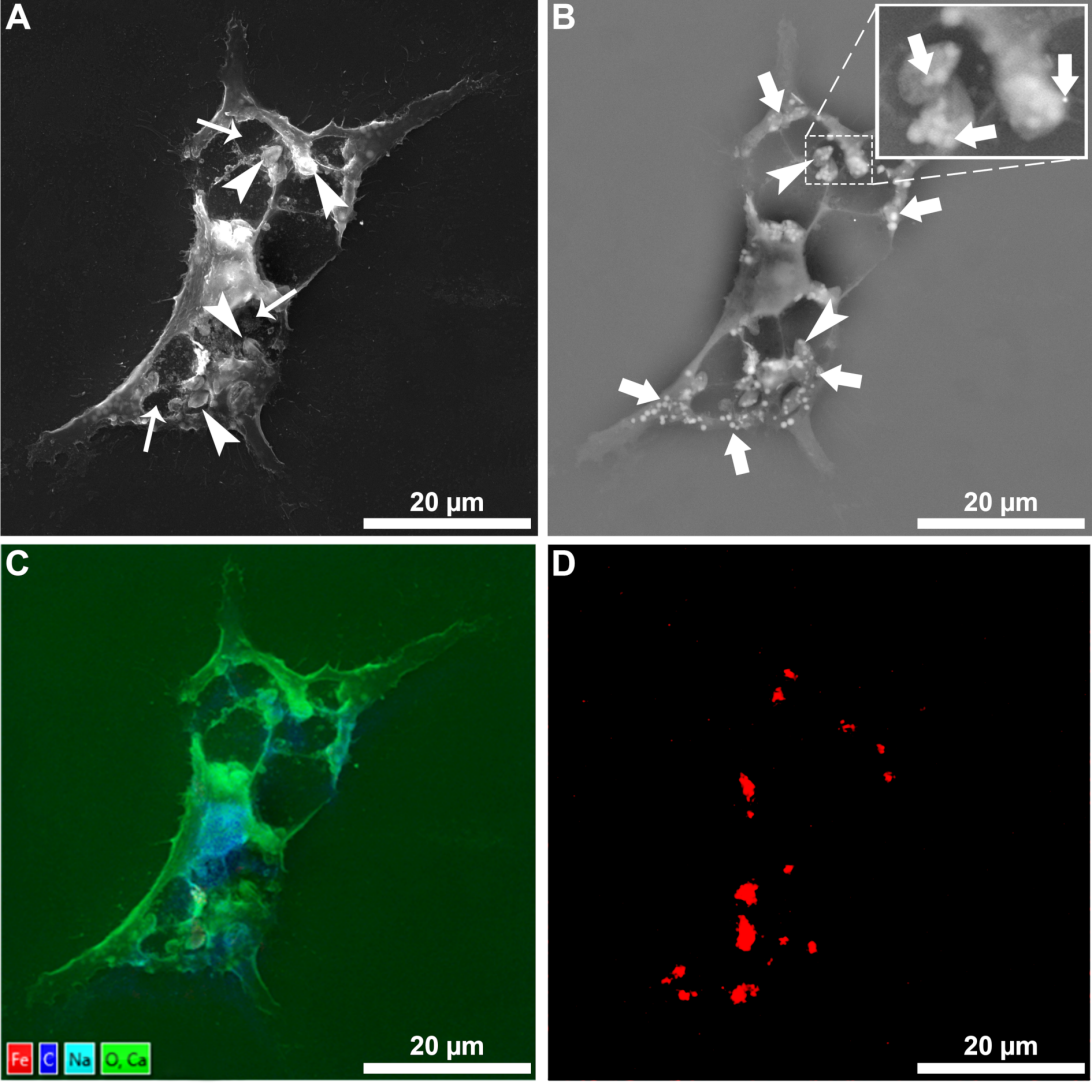
Scanning electron microscopy of macrophages infected with *L. amazonensis* intracellular amastigotes after treatment with 100 µg/mL SPIONs for 24 h. The plasma membrane was gently removed to observe the presence of nanoparticles inside the cells. Panel A shows infected macrophages with some amastigotes (arrowheads) inside the parasitophorous vacuoles (thin arrows). Panel B shows the same macrophage; however, the image was obtained by detecting backscattered electrons, revealing several electron-lucent aggregates (arrows). Digital magnification (highlighted rectangular area) showed electron-lucent aggregates even inside intracellular amastigotes (arrowheads). Panels C and D show the X-ray microanalysis mapping of infected macrophages, indicating the presence of iron in the cytosol (red color in Figure 2D).

After the first microscopic analysis, scanning electron microscopy and chemical element mapping analysis were carried out to confirm the uptake of the SPIONs by *L. amazonensis* intracellular amastigotes after removing the plasma membrane to expose the cytoplasmic environment (Figure 2). Secondary electron imaging revealed intracellular amastigotes inside the parasitophorous vacuoles (Figure 2A). Backscattered electron imaging showed several small electron-lucent structures randomly distributed throughout the macrophage cytosol, inside the parasitophorous vacuoles (Figure 2B, arrows), and in the intracellular amastigotes (Figure 2B, arrowheads). The ferrous nature of the observed structures was assessed by chemical element mapping analysis using energy-dispersive X-ray spectroscopy (Figure 2C), confirming that the electron-lucent structures contain iron atoms (Figure 2D).

**Figure 3:**
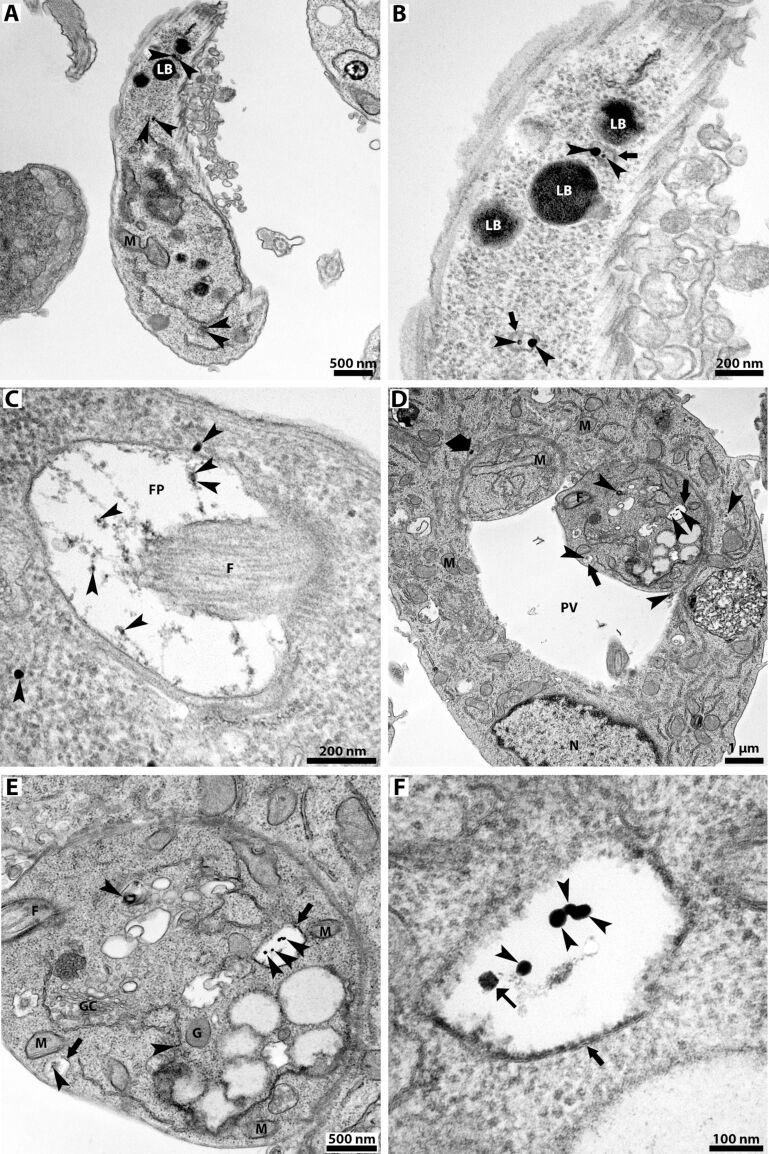
Transmission electron microscopy of *L. amazonensis* promastigotes and intracellular amastigotes treated with 100 µg/mL of SPIONs for 24 h. Electron-dense aggregates of SPIONs (arrowheads) are randomly distributed in both developmental stages. (A) SPIONs (arrowheads) were observed in the promastigote cytosol, closely associated with endoplasmic reticulum profiles and lipid bodies. (B) High-magnification image with SPION aggregates (arrowheads) inside membrane-bound compartments (arrows). (C) SPIONs (arrowheads) are associated with thin filaments inside the flagellar pocket and in the cytosol closely associated with the flagellar pocket membrane. (D) In the macrophages infected with intracellular amastigotes, the SPIONs appear inside the parasitophorous vacuole and in the macrophage and parasite cytosol (arrowheads). In this image, it is also possible to observe the SPIONs surrounded by a membrane (arrows) and an aggregate close to the membrane of the parasitophorous vacuole (large arrow). (E, F) High-magnification images of intracellular amastigotes revealing SPIONs (arrowheads) inside membrane-bound compartments (arrows). The aggregates are formed by smaller individual nanoparticles (small arrow). Figure 3E also shows many lipid bodies, vacuoles, and a multivesicular structure, which are features typically found in treated parasites. F, flagellum; FP, flagellar pocket; LB, lipid body; M, mitochondrion; N, nucleus; and PV, parasitophorous vacuole.

Transmission electron microscopy (TEM) was used to confirm the internalization of the SPIONs. First, promastigotes were treated with 100 µg/mL of SPIONs for 24 h (Figure 3A–C). TEM images confirmed the presence of SPION aggregates randomly distributed throughout the cytoplasm of the promastigotes (Figure 3A–C, arrowheads). The images suggest that these aggregates have different sizes. Furthermore, at high magnification, it is possible to observe that the SPIONS are frequently surrounded by membranes (Figure 3B, arrows). In addition, SPIONs were also observed inside the flagellar pocket (Figure 3C, arrowheads) and closely associated with the membrane.

The uptake of SPIONs was also observed in macrophages infected with *L. amazonensis* intracellular amastigotes after treatment with 100 µg/mL of SPIONs for 24 h (Figure 3D–F). The images confirmed the presence of SPION aggregates inside the macrophage cytosol, the parasitophorous vacuoles, and the intracellular amastigotes (Figure 3C,D, arrowheads). SPIONs were also observed inside the macrophages close to the parasitophorous vacuole membrane (Figure 3D, large arrow), sometimes appearing inside membrane-bound structures and exhibiting different sizes (Figure 3E, arrowheads). Some alterations in amastigote ultrastructure can also be observed, namely electron-lucent lipid bodies, a multivesicular body close to the Golgi complex, and endoplasmic reticulum profiles very close to organelles such as mitochondrion and glycosome. Higher magnification revealed that the SPION aggregates are constituted of small nanoparticles that appear associated with tiny filaments (Figure 3F, thin arrow).

### Antiproliferative effects of SPIONs in *L. amazonensis* promastigotes and intracellular amastigotes

**Figure 4:**
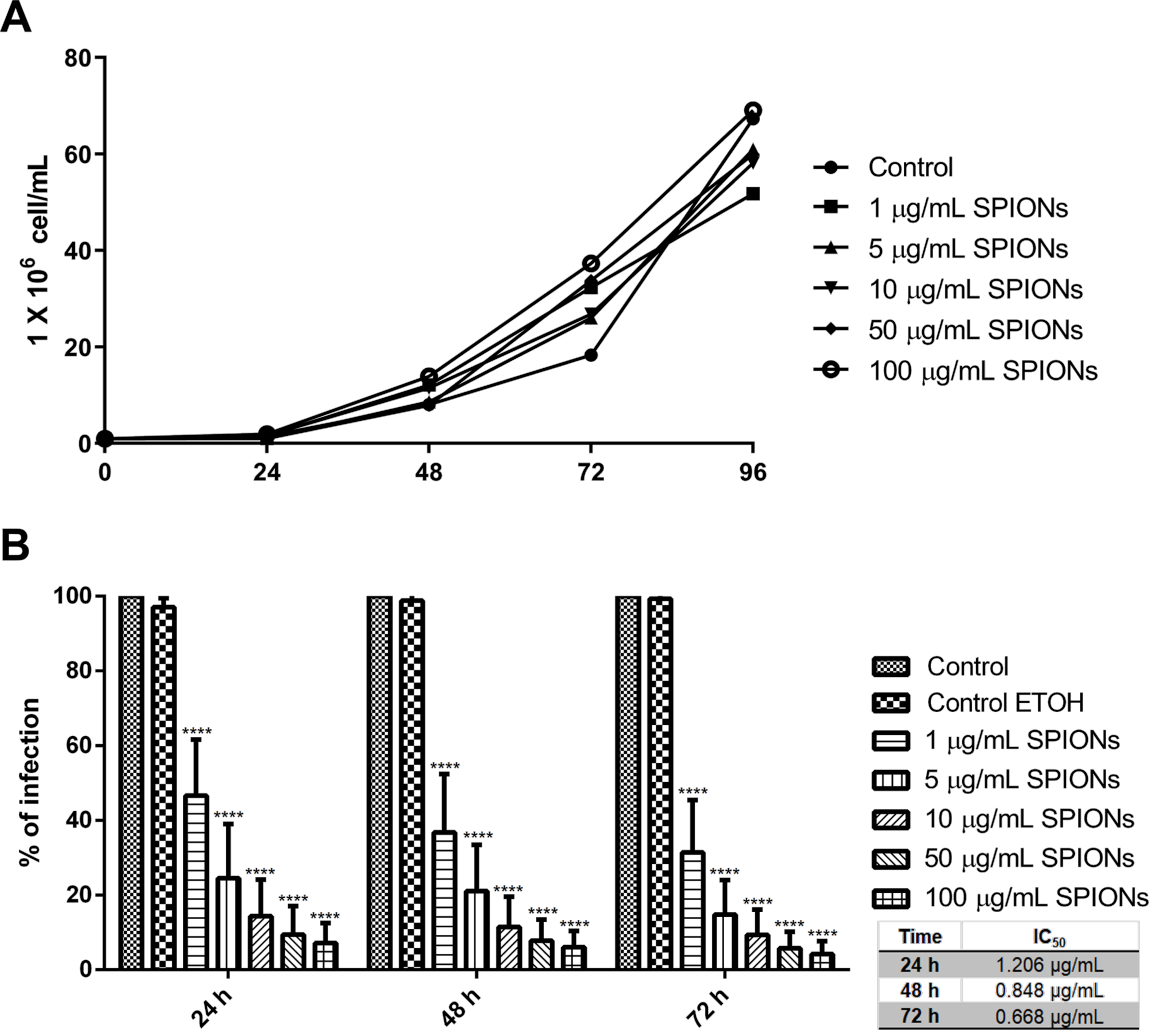
Analysis of the antiproliferative effect in *L. amazonensis* promastigotes and intracellular amastigotes treated with different concentrations of SPIONs. (A) Growth curve of *L. amazonensis* promastigotes; the SPIONs were added to the culture medium after 24 h of growth. (B) For intracellular amastigotes, infected macrophages were treated, and the percentage of infection was obtained for each treatment condition; the SPIONs were added to the infected macrophage culture after 24 h of infection. *P* values for panel B: **** *p* < 0.0001.

The analysis of the antiproliferative effects of SPIONs in *L. amazonensis* promastigotes showed that they could not alter the growth for any of the concentrations evaluated (Figure 4A). In contrast, the SPIONs were very active against intracellular amastigotes (Figure 4B). Furthermore, analysis of the growth curve shows a statistically significant reduction in the percentage of infection for all tested concentrations of SPIONs (1, 5, 10, 25, and 50 µg/mL) and treatment times (24, 48, and 72 h) when compared with the control of infected macrophages.

After the first 24 h of treatment, it was possible to observe a reduction in the percentage of infection of about 50% for a concentration of 1 µg/mL and of about 90% for 50 µg/mL of SPIONs. The data revealed a concentration-dependent effect, which increased within 48 and 72 h of treatment. The percentage of infection significantly reduces over time, indicating a time-dependent effect. The IC_50_ values were calculated for each treatment time and confirmed the results obtained (Figure 4B), that is, 1.206, 0.848, and 0.668 µg/mL for treatment times of 24, 48, and 72 h, respectively.

### Evaluation of possible effects on the ultrastructure of *L. amazonensis* intracellular amastigotes

**Figure 5:**
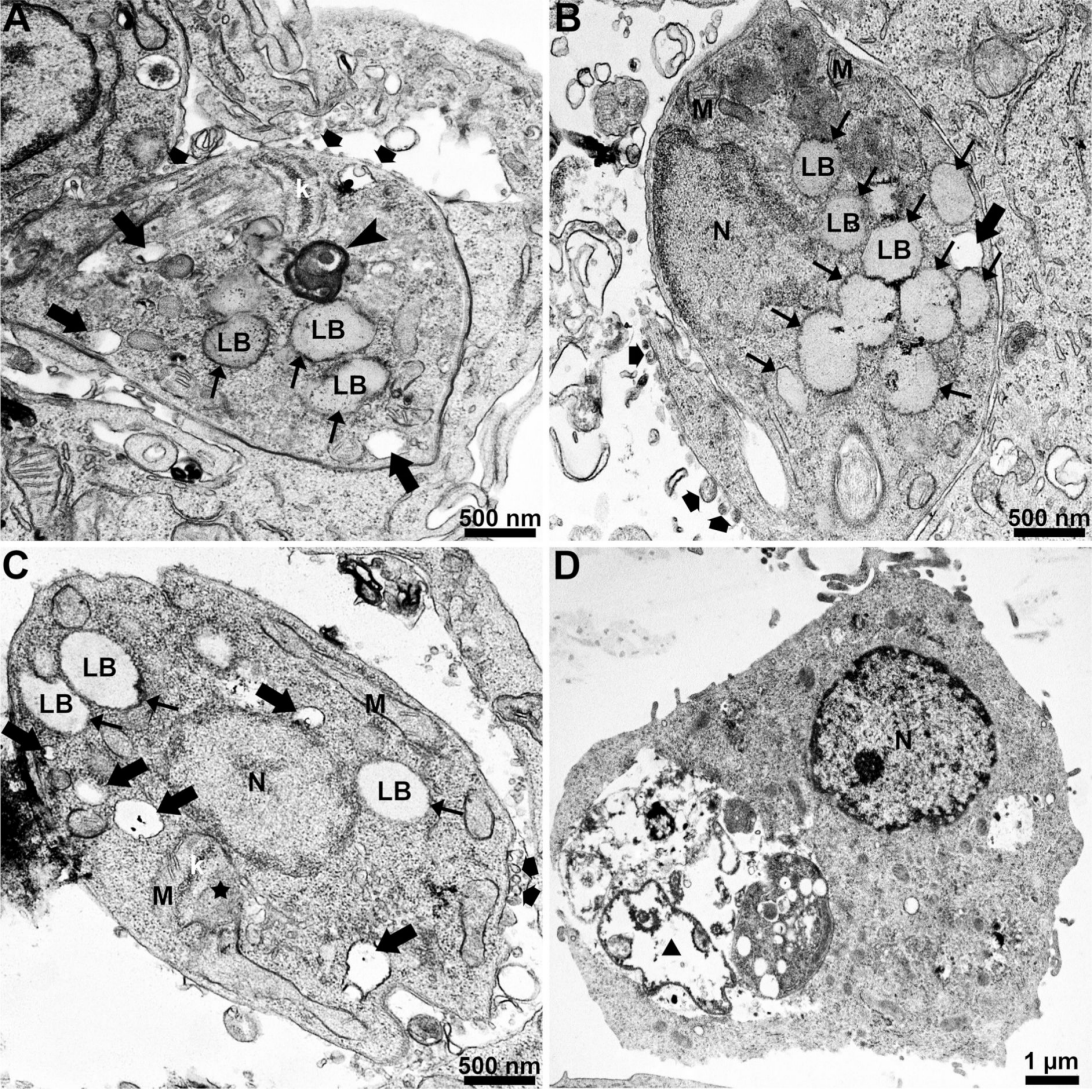
Transmission electron microscopy of *L. amazonensis* intracellular amastigotes treated with 100 µg/mL of SPIONs for 24 h. Different ultrastructural changes were observed in intracellular amastigotes: (1) many lipid bodies (A–C, thin arrows), (2) increased secretion of extracellular vesicles (A–C, broad arrows), (3) intracellular vacuolization (A–C, arrows), (4) myelin-like figures (A, arrowhead), (5) mitochondrial swelling (C, star), and (6) destroyed amastigotes (D, triangle). F, flagellum; k, kinetoplast; LB, lipid body; M, mitochondrion; and N, nucleus.

Transmission electron microscopy allowed us to analyze ultrastructural alterations induced by treating *L. amazonensis* intracellular amastigotes with 100 µg/mL of SPIONs for 24 h (Figure 5). The images revealed several alterations, namely (1) lipid bodies (Figure 5A–C, thin arrows), (2) cytoplasmic disorganization with many vacuoles, which may indicate activation of autophagic processes (Figure 5A–C, arrows), (3) myelin-like figures (Figure 5A, arrowhead), and (4) mitochondrial swelling (Figure 5C, star). Furthermore, in the intracellular amastigotes, there are membrane-bound compartments containing SPION aggregates and parasitophorous vacuoles containing cellular debris and dead amastigotes (Figure 5D, triangle).

## Discussion

SPIONs represent a new approach to diagnosing and treating diseases, particularly when associated with magnetic hyperthermia, an emerging form of active treatment [14–18]. However, despite all their potential, the synthesis processes of the SPIONs are characterized by being expensive and toxic to humans and the environment [6]. In this scenario, our group demonstrated the therapeutic potential of low-cost biocompatible SPIONs produced by green synthesis [9]. The present study aimed to evaluate in vitro the therapeutic potential of SPIONs produced with coconut water to treat cutaneous leishmaniasis caused by *L. amazonensis*.

Microscopy techniques efficiently revealed the uptake and distribution of SPIONs in *L. amazonensis* promastigotes and intracellular amastigotes. The first analysis confirmed the uptake of SPIONs by macrophages, which was published previously by our group [9]. Furthermore, in the article here, the images revealed SPIONs inside the parasitophorous vacuole and in the cytosol of intracellular amastigotes. In addition, SPIONs were also observed randomly distributed throughout the cytosol of promastigotes, in the flagellar pocket, and inside membrane-bound structures. It is the first time that superparamagnetic iron oxide nanoparticles SPIONs are observed inside the *Leishmania* spp and the parasitophorous vacuole. Chemical element mapping analysis by scanning electron microscopy confirmed the ferrous nature of the nanoparticle aggregates. These results prove the ability of both promastigotes and intracellular amastigotes to uptake SPIONs from the culture medium.

The acquisition of iron by *Leishmania* intracellular amastigotes that live inside mammalian host cells is important for cell differentiation and the pathogenesis of the disease [19–21]. Thus, it is possible to speculate that SPIONs use iron transport mechanisms to reach the parasitophorous vacuole and amastigote cytosol [21]. However, further studies need to be carried out to confirm this hypothesis and to elucidate the mechanisms of SPION uptake in promastigotes and amastigotes.

We evaluated the antiproliferative effects of SPIONs in *L. amazonensis* promastigotes and intracellular amastigotes. Despite being internalized by promastigotes, SPIONs did not affect the cell proliferation of the parasites (Figure 4A). A completely different result was observed for intracellular amastigotes, where the reduction in the percentage of infection was very significant already with the lowest concentration of SPIONs used [1 µg/mL] (Figure 4B). The IC_50_ values found for intracellular amastigotes during the treatment were 1.206, 0.848, and 0.668 µg/mL for treatment times of 24, 48 and 72 h, respectively. In a previous study published by our group, we analyzed the cytotoxicity of SPIONs against macrophages [9]. The results revealed no toxic effects up to a concentration of 300 µg/mL, indicating that SPIONs are well tolerated by macrophages. Because CC_50_ values are difficult to calculate, we used GraphPad Prism software to estimate them. CC_50_ values are essential to calculate the selective index (SI), and both quantities are important to understand how effective the nanoparticles are against the parasite while being less toxic for mammalian cells (Table 1).

**Table 1:** Estimated CC_50_ and SI obtained after the analysis of the macrophage cytotoxicity assay previously published in [9] using the GraphPad Prism software.

Time	Estimated cytotoxic concentration of 50% (CC_50_) for macrophages	Estimated selective index (SI)

24 h	1271.5 µg/mL	1054
48 h	2250.6 µg/mL	2654
72 h	3420.0 µg/mL	5119

The SI revealed that the SPIONs were highly selective for *L. amazonensis* intracellular amastigotes (Table 1), presenting values significantly higher when compared with other compounds and drugs used to treat *Leishmania* sp. (Table 2) [22–27]. These data indicate a high selectivity index for SPIONs compared with current treatments, different from most compounds, drugs, and nanomaterials developed in the last decades.

**Table 2:** Selectivity index values for different compounds and drugs studied and used for treating leishmaniasis.

Time	Compound	SI	Reference

24 h	amphotericin B	16	[26]
48 h	TC95	24	[23]
48 h	KH-TFMDI	81	[22]
72 h	itraconazole	103.17	[25]
72 h	ravuconazole	28.9	[24]
72 h	miltefosine	34.2	[27]

During TEM analyses, we observed that intracellular amastigotes were undergoing substantial ultrastructural alterations (Figure 5) when treated with SPIONs. These alterations include (1) accumulation of lipid bodies, (2) intense intracellular vacuolization, (3) mitochondrial swelling, (4) myelin-like figures, and (5) cell death. The observed ultrastructural effects corroborate the significant antiproliferative effect found and give indications of the possible mechanisms of action of these nanoparticles, which may be closely associated with intracellular iron homeostasis.

Iron homeostasis has been extensively studied because of its essential role in maintaining the cellular functions of several cell types. It is well established that, in mammalian cells, iron in its free state can participate in the Haber–Weiss reaction, catalyzing the formation of highly reactive hydroxyl radicals that lead to oxidative stress [28–29]. Thus, one of the possibilities for the observed antiproliferative effects could be the result of an imbalance in iron homeostasis with the consequent induction of oxidative stress and death of the parasites. However, further studies need to be carried out to confirm this hypothesis. In *Leishmania,* it is well known that available iron has an important influence on the homeostasis of reactive oxygen species [30]. Studies have already shown that iron excess in the diet of mice causes a decrease in the replication of *Leishmania* spp. in different tissues of infected animals due to the interaction with reactive oxygen and nitrogen species [31–32].

Several studies have shown the potential of using nanoparticles as a new method for treating leishmaniasis. However, only a few studies report the effects of using iron oxide nanoparticles [11–12,15,33–35]. Recently, the effects of magnetic iron oxide nanoparticles were demonstrated in *L. mexicana* axenic amastigotes. First, the amastigotes were treated with 200 µg/mL of magnetic nanoparticles. Subsequently, magnetic hyperthermia was applied using an alternating field of 30 mT with a frequency of 452 kHz for 40 min. The results showed that magnetic hyperthermia was efficient in killing *L. mexicana* axenic amastigotes [12]. Another study demonstrated the anti-*Leishmania* effect of magnetic nanoparticles synthesized by green chemistry in *L. major* promastigotes [35]. Finally, a study showed the effect in vitro and in vivo of amphotericin B encapsulated in magnetic iron oxide nanoparticles coated with glycine-rich peptides for treating visceral leishmaniasis caused by *L. donovani* [12]. All these studies demonstrated the potential gain of drug conjugation with magnetic nanoparticles for treating leishmaniasis.

## Conclusion

The use of SPIONs synthesized with coconut water to treat macrophages infected with *Leishmania amazonensis* intracellular amastigotes revealed a significant anti-*Leishmania* effect with a selectivity index more than 240 times higher than those of other currently used drugs. Furthermore, it was also observed that the SPIONs could be directed into the parasitophorous vacuoles of infected cells and parasites. Thus, this new nanomaterial is a promising new therapeutic alternative as (1) an active treatment agent because of its intrinsic properties, (2) a treatment agent associated with heating through alternating current magnetic fields, and (3) a drug carrier.

Finally, SPIONs can be considered a strong candidate for a new therapeutic approach to treating cutaneous leishmaniasis, that is, an accessible and low-cost topical treatment.

## Experimental

### SPIONs

The SPIONs used in the present study were synthesized as described in [9] (patent application registration BR 10 2020 015814 [36]). For assays, after synthesis and purification, the SPIONs were dispersed in a 70% ethanol solution (Merck, Germany). The maximum ethanol concentration in cultures did not exceed 0.5%, which did not interfere with cell growth. The nanoparticles used in the biological tests were stored at −20 °C.

### Ethics committee for the use of laboratory animals

The assays that used mammalian macrophages and parasites from animal models were approved by the Ethics Committee for the Use of Laboratory Animals (CEUA) of the Centro de Ciências da Saúde from the Universidade Federal do Rio de Janeiro according to the Brazilian Federal Law (11794/2008, Decreto No. 6,899/2009). For the use of peritoneal macrophages resident in mice and the maintenance of *Leishmania amazonensis* species in Balb/C mice, the protocol number was UFRJ/CCS-142/21. Furthermore, all animals received human care according to the guide published by the Brazilian Society of Zootechnics of Laboratory and Council National Control of Animal Experimentation.

### Cell culture

The immortalized murine macrophages RAW 264.7 were grown in 25 cm^2^ bottles in RPMI 1640 medium (Cultilab, Brasil) supplemented with 2% sodium bicarbonate, 10% fetal bovine serum, and 100 U/mL penicillin. Cells were cultured at 37 °C in 5% CO_2_ atmosphere, and the medium was changed three times a week; cells were passed when they reached confluence in the bottles. In addition, primary cultures of murine macrophages were obtained from the peritoneal cavity of CF1 mice by washing with Hanks’ balanced solution. Then, they were plated on coverslips in a 24-well culture plate and placed to adhere for 24 h at 37 °C in an atmosphere of 5% CO_2_. For the microscopic analyses, macrophages were grown in 25 cm^2^ bottles or on glass coverslips in 24-well plates; after 24 h of culture, they were treated for 24 h with different SPION concentrations. This study used the WHOM/BR/75/JOSEFA *Leishmania amazonensis* strain as a standard model for cutaneous leishmaniasis. The parasites were maintained according to previously published protocols [22].

### Prussian blue staining

For staining with Prussian blue (Sigma-Aldrich, Germany), promastigote and intracellular amastigotes were treated with 100 µg/mL of SPIONs for 24 h. The promastigotes (control and treated cells) were washed in phosphate-buffered saline (PBS) pH 7.2 and adhered for 10 min on glass coverslips previously coated with poly-ʟ-lysine (Sigma-Aldrich, Germany). The intracellular amastigotes were obtained after infection of RAW 264.7 macrophages at a ratio of ten parasites to one macrophage. After treatment, cells were washed in PBS pH 7.2, fixed, and dehydrated, as described in [9]. Finally, cells were observed using a DM2500 optical microscope (Leica Microsystem, Germany) in bright-field mode.

### Electron microscopy analysis

Control and treated cells were washed in PBS pH 7.2, fixed, and post-fixed according to previously published protocols [23]. Then, cells were processed for scanning electron microscopy and chemical element mapping analysis as described in [9]. The micrographs were obtained using a TESCAN VEGA 3 LMU scanning electron microscope operating at 20 kV equipped with an OXFORD X-MaxN 20 mm^2^ detector (Oxford Instruments, United Kingdom) for energy-dispersive X-ray spectroscopy. For transmission electron microscopy, after fixation, samples were dehydrated in increasing acetone concentrations and embedded in Epon. Ultrathin sections were obtained using a PT-PC PowerTome ultramicrotome (RMC Boeckeler, USA) stained with uranyl acetate and lead citrate and observed using a FEI TECNAI SPIRIT transmission electron microscope operating at 120 kV.

### Antiproliferative effects of SPIONs in *Leishmania amazonensis* promastigotes and intracellular amastigotes

To evaluate the effect of the SPIONs on the growth of *L. amazonensis* promastigotes, cell density experiments were initiated with an inoculum of 1.0 × 10^6^ parasites/mL in M199 culture medium supplemented with 10% fetal bovine serum and cultivated at 25 °C. After 24 h of growth, different concentrations of SPIONs (1, 5, 10, 50, and 100 µg/mL) were added, and cells were cultured for 96 h. The cell density was calculated every 24 h by counting the number of cells in a Neubauer chamber using contrast-phase light microscopy. Besides, SPIONs were also evaluated against intracellular amastigotes, the clinically relevant stage of leishmaniasis. For this analysis, murine macrophages and parasites were obtained as previously published [23]. After 24 h of the initial infection, different concentrations of SPIONs (1, 5, 10, 25, and 50 µg/mL) were added, and the medium with the nanoparticles was changed every day for three days. The IC_50_ was calculated using the linear regression method defined in [37].

### Statistical analysis

Statistical analysis was conducted using GraphPad Prism with one-way analysis of variance (ANOVA). The results were considered statistically significant for cases of *p* ≤ 0.05 (*).
